# Transducer Development and Characterization for Underwater Acoustic Neutrino Detection Calibration

**DOI:** 10.3390/s16081210

**Published:** 2016-08-02

**Authors:** María Saldaña, Carlos D. Llorens, Ivan Felis, Juan Antonio Martínez-Mora, Miguel Ardid

**Affiliations:** Institut d’Investigació per a la Gestió Integrada de les Zones Costaneres (IGIC), Universitat Politècnica de València (UPV), 46730 Gandia, Spain; cdavid@upv.es (C.D.L.); ivfeen@upv.es (I.F.); jmmora@fis.upv.es (J.A.M.-M.)

**Keywords:** acoustic calibrator, piezo-ceramic tube transducers, Ultra-High Energy neutrinos, acoustic detection, underwater neutrino telescopes, parametric technique

## Abstract

A short bipolar pressure pulse with “pancake” directivity is produced and propagated when an Ultra-High Energy (UHE) neutrino interacts with a nucleus in water. Nowadays, acoustic sensor networks are being deployed in deep seas to detect this phenomenon as a first step toward building a neutrino telescope. In order to study the feasibility of the method, it is critical to have a calibrator that is able to mimic the neutrino signature. In previous works the possibility of using the acoustic parametric technique for this aim was proven. In this study, the array is operated at a high frequency and, by means of the parametric effect, the emission of the low-frequency acoustic bipolar pulse is generated mimicking the UHE neutrino acoustic pulse. To this end, the development of the transducer to be used in the parametric array is described in all its phases. The transducer design process, the characterization tests for the bare piezoelectric ceramic, and the addition of backing and matching layers are presented. The efficiencies and directivity patterns obtained for both primary and parametric beams confirm that the design of the proposed calibrator meets all the requirements for the emitter.

## 1. Introduction

Astrophysical neutrino detection is based on Cherenkov light measurement induced by secondary leptons, like muons, which are produced by neutrino interactions with matter while passing across the Earth. Ongoing searches for cosmic ray neutrinos endeavor to detect muons moving upward from the Cherenkov light in either ice or water. The IceCube telescope, located at the South Pole, has recently discovered high-energy astrophysical neutrinos [1] boosting the neutrino astronomy field. Besides IceCube, the existing neutrino telescopes currently in use are the NT200+ in Lake Baikal [2], ANTARES [3] and a new optical-based deep-sea neutrino telescope under construction, the KM3NeT telescope [4], which will have a volume of several cubic kilometres.

Ultra-High Energy (UHE) neutrinos (~10^20^ eV) have been of great interest for the study of ultra-high energy cosmic ray sources and to test fundamental physics due to being high-energy, stable and weakly interacting elementary particles. Besides Cherenkov light, radio or acoustic wave techniques were proposed to detect the neutrino-induced energy deposition in water, ice or salt. These techniques, with much longer attenuation lengths, allow for very large target volumes utilizing either large ice fields or dry salt domes for radio or ice fields, and the oceans for acoustic detection.

Acoustic detection of UHE neutrinos is based on the thermo-acoustic effect [5]. When an UHE neutrino interacts with a nucleus in water, its energy is released in a volume of about a centimetre in radius and several meters in length. This phenomenon induces a local heating in a very short period of time leading to a short pressure pulse signal with bipolar shape in time and a very directive pattern (pancake-like), being emitted mainly in the perpendicular plane of the shower axis [6].

Over the last few decades there have been a number of experiments looking into acoustic neutrino detection in both water and ice media, such as AMADEUS [7], SPATS [8], ACORNE [9] and SAUND [10]. The detection technique is still under study and could be implemented in a new optical neutrino telescope KM3NeT [11]. The acoustic detection would allow the combination of those two neutrino detection techniques for a hybrid underwater neutrino telescope, especially considering that the optical neutrino detection technique needs acoustic sensors to monitor the position of the optical sensors [12].

Therefore, an emitter able to imitate the bipolar pulse signal generated by the neutrino in water will be extremely useful. For this purpose, an acoustic array calibrator is being designed. The emissions from the emitter calibrator, which will be time controlled, will allow the sensors to be trained for neutrino detection. Furthermore, it will improve the classification and identification of the acoustic neutrino signals, telling them apart from noise or other transient background signals [13].

The acoustic calibrator will be deployed in the vicinity of the detector with a distance between the emitter calibrator and the detector of 0.5 to 3.5 km. The furthest distances would occur when the calibrator is operated from the sea surface, and the closest when operated from the seabed. Consequently, the acoustic calibrator needs to be quite powerful in order to reach the detector with enough pressure to be detected and recognized.

The objective of the acoustic array calibrator is the emission of bipolar pulse signals with similar characteristics to the signal produced by a 10^20^ eV neutrino interacting in water at a distance of 1 km, which would be detected with an amplitude of about 10 mPa in the low-ultrasonic frequency range (maximum amplitude between 5 kHz and 20 kHz) with an opening angle of about 1°. The proposed design for the acoustic calibrator is a compact array system composed of piezo-ceramic tube transducers emitting in the radial direction. The emission is amplified and controlled by specific electronics adapted to them. The emission of the low-frequency (tens of kHz) acoustic bipolar pulse is generated by using the parametric emission technique at a high frequency (hundreds of kHz).

## 2. Compact Array Calibrator Based on the Parametric Acoustic Source Technique

The use of the parametric acoustic source technique to generate neutrino-like signals allows the generation of low-frequency signals with narrow directivity, which is essential in order to obtain bipolar signals with the required “pancake” directivity. This technique was already validated in previous studies using cylindrical transducers [14]. The acoustic parametric effect occurs when two intense monochromatic beams with two close frequencies travel together through the medium. According to the linear theory, and as a consequence of the principle of superposition, the resulting sound field is composed only of the initial frequencies. However, due to the non-linearity of the medium, the interaction of the two frequency beams of finite amplitude generates a set of secondary frequency beams, such as the sum and difference of the primary frequency signals. This process of non-linear generation of new frequencies is limited to a certain distance from the transducer, called the array length, given by the distance of interaction or absorption length. This may be considered as a set of virtual acoustic sources (array) contained along the length of interaction; the source seems to be shaded exponentially as the distance increases from the transmitter. The secondary beam is produced with a directivity pattern similar to the one of the primary beams, which offers the advantage of generating a low-frequency directive beam.

**Figure 1:**
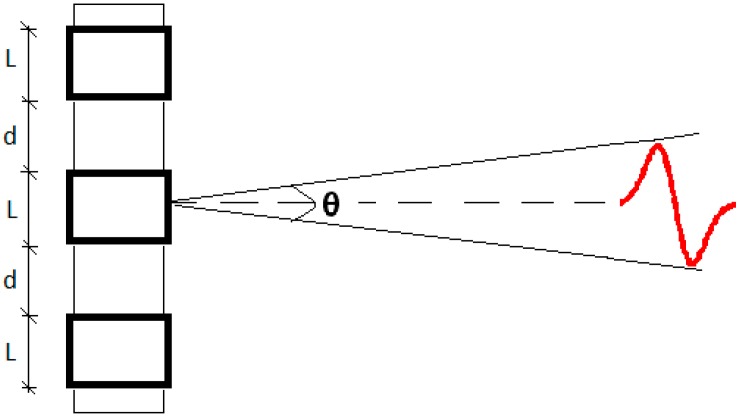
Schematic diagram of the transducer array configuration with 3 elements.

The parametric technique for the emission of the low-frequency signal previously cited allows us to design a compact array consisting of fewer units with respect to classical solutions. Moreover, this technique will reduce costs and facilitate the deployment and operation of the calibrator. The proposed array calibrator under development will be constituted of three to five elements of the piezo tube ceramic structured in the same axis-line, with a distance separation (d) between 10 cm and 20 cm, in order to obtain an opening angle of about 1° with phased emission of the array components. The transducer array will emit in radial direction and produce a bipolar pulse due to the interaction of parametric signals generated by each array element. Figure 1 shows a schematic diagram of the configuration and emission.

**Figure 2:**
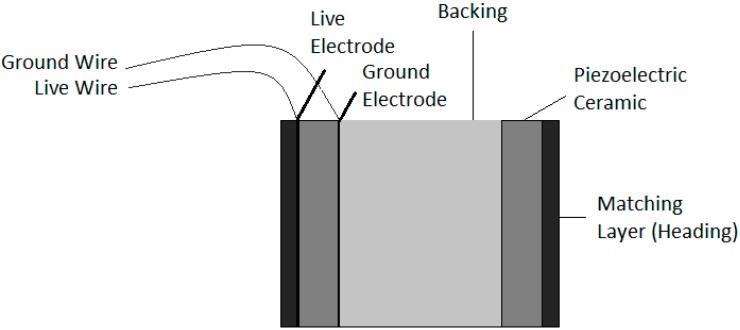
Drawing of the transducer design component, ceramic, backing and matching layer.

In order to increase the functionality of the calibrator and facilitate the calibration under different circumstances, the proposed calibrator is designed to work in different operation modes. The three operation modes are: (1) at linear low frequency range by emitting long non-directive signals (easy to generate and easy to detect); (2) at high frequency range by emitting long parametric directive signals (signal processing techniques could be used to simplify detection of these signals); and (3) at high frequency range by emitting the transient and directive parametric bipolar signal (the most challenging case). These operation modes will enable the array transmitter to achieve different goals, such as training and tuning the acoustic detector, cross-checking the detector hydrophones, and other marine applications. The proposed calibrator design and development was divided into three phases. In the first stage, the study and selection of transducers was done. Once the transducers were selected, their characterization was performed and measures with the single bare transducer emitting parametric neutrino-like signals were realized. In the second phase, the study and selection of backing and matching layer materials were carried out for optimizing the ceramic emission, and for holding and isolating the ceramics as well. As a result, a final prototype transducer (backed and moulded) convenient for this application was selected. Figure 2 shows a diagram of the transducer design with the backing and matching layer configuration. This paper is focused on these two phases. For future research, a third phase is planned and it will be focused on the design of the complete array system composed of a few units of the developed transducer. In this future phase, electronics will be adapted to the array emitters in order to amplify the power of the signal emission, as well as to include all the functionalities for the control and operation of the calibrator. Once completed, long distance and in situ tests will be performed in the detector vicinity.

## 3. Transducer Selection and Characterization

**Figure 3:**
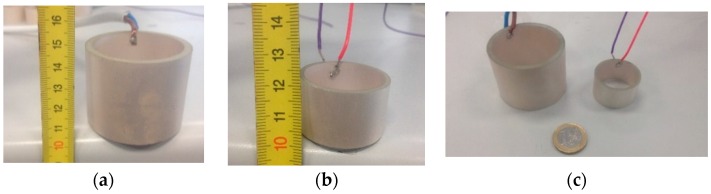
(**a**) Piezo-ceramic large tube; (**b**) Piezo-ceramic small tube; (**c**) Both piezo-ceramics.

**Figure 4:**
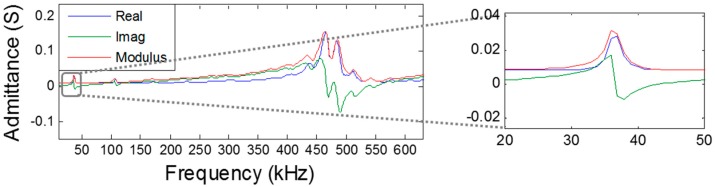
Admittance of the large tube.

Two commercial piezo-ceramic tube transducers were selected as emitter candidates of the array calibrator. The selection was made in terms of resonance frequencies, power emission, dimensions, and costs. Both transducers permit high-frequency signal emissions with high power, exhibiting reasonable power levels at low frequency as well. The first candidate was the piezo-ceramic UCE-534541, hereinafter referred to as the large tube. Its dimensions are: outer diameter, 5.3 cm; inner diameter, 4.5 cm; and height, 4.1 cm. The large tube primary resonance frequency is around 490 kHz with a real impedance of 9 Ω, with a secondary resonance frequency at low frequency, around 35 kHz. The second candidate is the piezo-ceramic UCE-343020, hereinafter referred to as the small tube. Its dimensions are: outer diameter, 3.4 cm; inner diameter, 3 cm; and height, 2 cm. The small tube primary resonance frequency is located around 890 kHz with a real impedance of 6 Ω, with a secondary frequency resonance around 75 kHz. Figure 3 shows a picture of both ceramics and Figure 4 shows the admittance of the large tube, where the resonance frequencies are appreciated.

### 3.1. Transmitting Voltage Response and Directivity

**Figure 5:**
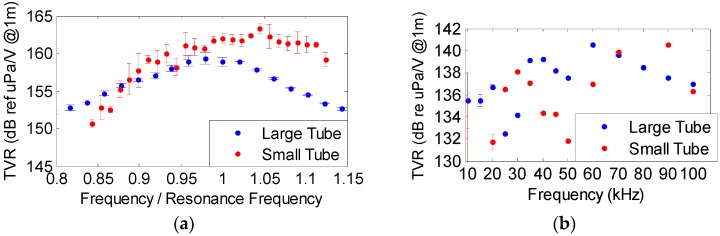
Characterization of the ceramics (**a**) TVR of large tube at F_R_ = 490 kHz (blue dots) and for small tube at F_R_ = 890 kHz (red dots); (**b**) TVR from 10 to 100 kHz.

**Figure 6:**
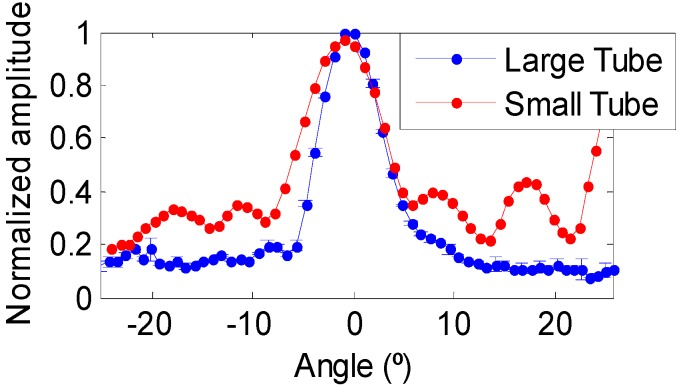
Directivity at 490 kHz for the large tube (blue) and at 890 kHz for small tube (red).

The piezo-ceramics were characterized both at high and low resonance frequencies in a water tank of 87.5 × 113 × 56.5 cm^3^ with fresh water at the laboratory. The calibration of the ceramics was performed in terms of the Transmitting Voltage Response (TVR), which is the ratio of the pressure signal emitted to the applied voltage, using tone bursts at different frequencies. The emission directivity with respect to the equatorial plane of the cylinder was also evaluated. The hydrophones used for these measurements are omnidirectional, particularly, model RESON-TC4038 for the high frequency range and the model RESON-TC4034 for the low frequency range. Figure 5 shows the characterization of both ceramics. The sensitivities obtained at high frequency with resonance frequency (F_R_) of 490 kHz for the large tube and 890 kHz for the small tube are shown in Figure 5a. The TVR of the large tube is 159 dB (re μPa/V at 1 m) at F_R_ = 490 kHz with a directivity of Full Width Half Maximum (FWHM) of 10° (Figure 6). At low frequencies, the TVR varies between 132 and 140 dB (re μPa/V at 1 m). On the other hand, the TVR of the small tube is 162 dB (re μPa/V at 1 m) at F_R_ = 890 kHz with a FWHM directivity of 14° (Figure 6). The TVR at low frequencies varies between 132 and 143 dB (re μPa/V at 1 m).

### 3.2. Backing

Some backing materials are able to absorb the energy produced from the back of the active element (ceramic) and reflect part of it forward, providing more acoustic power in emission [15]. In addition, the backing element of the transducer will constitute the support of the complete future array calibrator, i.e., playing an important role in the mechanical design of the array (Figure 1). Since all elements of the array are identical and emit synchronously it is expected that the coupling effect of the backing will be negligible.

**Figure 7:**
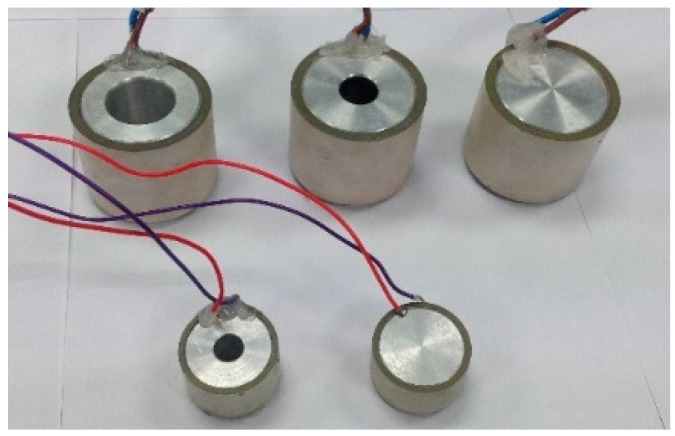
Piezo-ceramics with aluminium backings of different thicknesses.

Different materials and thicknesses were studied in order to find the best option in terms of TVR and acoustic impedance. The materials studied as backings were epoxy and aluminium. Figure 7 shows the ceramics implemented with the backing elements studied.

According to the results obtained on the characterization of all backings for both ceramic types, the set of aluminium backing completely filling the tube ceramic yielded the best results. For both ceramics the filled aluminium backing produced a flatter frequency response from the TVR. In the case of the backed large tube a 3 dB increment on the TVR, a resonance frequency shifting to 510 kHz, and an increment up to 30 Ω (real) in the impedance was observed. Regarding the small tube, an increase of 5 dB on the TVR, a shift on the resonance frequency to 950 kHz, and an impedance increment to 50 Ω (real) was appreciated. Moreover, there was not any appreciable change in the directivity patterns of both tubes.

### 3.3. Matching Layer

**Figure 8:**
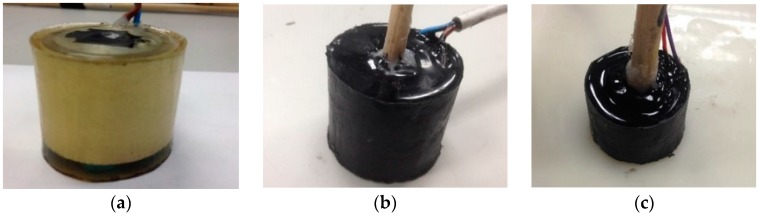
(**a**) Piezo-ceramic large tube with aluminium backing and RoyaPox 511 moulding; (**b**) Piezo-ceramic large tube with aluminium backing and polyurethane EL241F moulding; (**c**) Piezo-ceramic small tube with aluminium backing and polyurethane EL241F moulding.

Two interesting materials for moulding underwater transducers were acoustically studied in order to determine the effect on the signal emitted. Ensuring protection, isolation and holding, and matching the impedance of the piezo-ceramic to the impedance media [16] are the reasons for moulding the bare ceramic. The matching materials under study were the resin RoyaPox 511 and the Polyurethane EL241F. Both materials exhibit interesting acoustic properties for matching the ceramic impedance to the media due to their specific acoustic impedance (Z), density (ρ) and sound speed (c_L_). There is maximum transmission at the layer thickness of (2*n* − 1)*λ*/4. Since for the first mode, *λ*/4, the obtained thickness values were too thin for an optimum covering, the matching layer thicknesses were set to accomplish the second mode with thickness 3*λ*/4 of the wavelength emitted at the ceramic resonance frequency. Such a selection offers the advantage of sufficient thickness to facilitate the covering and obtaining maximum length transmission. The matching layer mould used was a cylinder with the thickness space necessary to cover the ceramic, controlled by a precise metric rule with an estimated uncertainty of ±0.1 mm.The acoustic properties of the materials and the thickness used are summarized in Table 1. In order to test both materials with the same ceramic, two large tubes with aluminium backing were moulded with each material. Figure 8 shows the ceramic covered with (a) RoyaPox 511 and (b) with polyurethane EL241F. The best results in terms of TVR and directivity were obtained with the polyurethane. Afterwards, a small tube with aluminium backing was moulded with polyurethane, as shown in Figure 8c.

**Figure 9:**
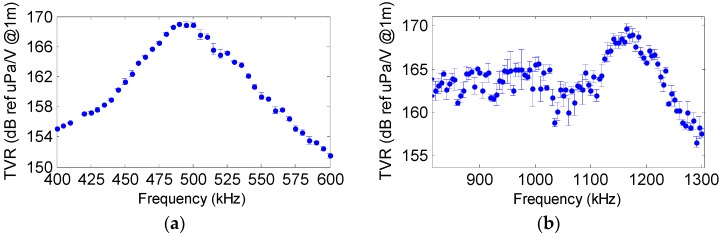
(**a**) TVR of large tube with aluminium backing and moulded with polyurethane EL241F; (**b**) TVR of small tube with aluminium backing and moulded with polyurethane EL241F.

The ceramic moulded with the RoyaPox 511 resin showed the same TVR level on the resonance frequency than before moulding. The resonance frequency shifted to 450 kHz, but the impedance and directivity were very similar to that obtained with the bare ceramic. For the case of the large tube moulded with the EL241F polyurethane, there was a TVR gain of 5 dB approximately in the resonance frequency compared to before moulding. The resonance frequency shifted to 495 kHz with a sensitivity of 169 dB (re μPa/V at 1 m). The TVR curve for this case is shown in Figure 9a. The impedance at the resonance frequency was 12 Ω (real), that is, it was reduced as compared to itself only backed, but being still higher than bare. With respect to the directivity, the pattern did not show any significant variation. Figure 9b shows the TVR of the small tube moulded with the polyurethane. The graph is practically the same as before moulding for the 890 kHz–1 MHz range. However, there was also a peak resonance at higher frequencies, for 1165 kHz with 169 dB (re μPa/V at 1 m) with real impedance of 20 Ω. Directivity pattern did not vary with respect to the bare one.

To sum up, the design with the aluminium backing and the moulding with polyurethane EL241F produced an increase on the TVR at resonance of 9 dB for the large tube and of 7 dB for the small tube. For both ceramics, the design did not change the directivity pattern of the ceramics.

## 4. Studies on Parametric Emission

The parametric acoustic source technique was evaluated by means of the low frequency-parametric generation of a sine sweep signal using the cross-correlation with the expected signal. Following this methodology, it is feasible to recognize the parametric signal, since the correlation produces a clear narrow peak on the signal arrival time, which allows it to be distinguished from near echoes, as well as an increase in the signal to noise ratio [17]. Afterwards, the low frequency-parametric generation of a bipolar shape pulse signal in time was studied. The generation of the portable signal for the bipolar pulse generation is explained in [14]. These portable signals for the parametric generation were implemented with modulation at high frequency, e.g., at the frequency resonance of the ceramic, either 495 kHz or 1165 kHz.

In order to confirm the non-linear effect of the parametric signal received three studies in the different aforementioned stages were conducted. The first study aimed at comparing the amplitude of primary and secondary beams by starting from low amplitude emission and increasing it to demonstrate the non-linear effect. The second measurement analysed the secondary non-linear beam generation in the medium by changing the distance between emitter and receiver. The last study compared the directivity patterns of both beams.

The experiment was done in the same water tank as described in Section 3.1. The piezo-ceramic was connected to a linear 55 dB (gain) RF amplifier ENI 1040L to feed the emitter and generate a more powerful signal in order to achieve the non-linear parametric effect. The receiver was the RESON-TC4034. This transducer is an omnidirectional, broad-band hydrophone with enough sensitivity to detect the primary beam (high frequency) even more sensitive to low frequency, i.e., for the bipolar pulse detection. Additionally, it was connected to a charge amplifier CCA 1000 (Teledyne RESON) which amplifies the received signal, especially for low frequency signals.

### 4.1. Parametric Sine Sweep Signal

**Figure 10:**
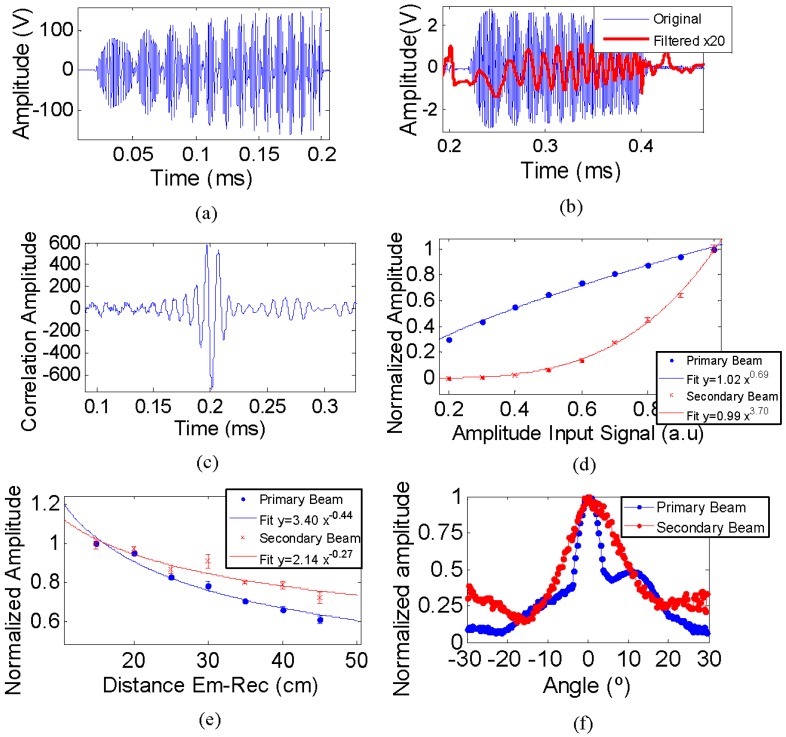
(**a**) Emitted signal for sine sweep (20 kHz–50 kHz) generation; (**b**) received signal (blue line) and the band-pass filtered signal (red line); (**c**) correlation of the original received signal with the expected secondary beam; (**d**) correlation amplitude of the received signal with primary beam (blue points) and secondary beam (red points) as a function of the signal emitted amplitude; (**e**) correlation amplitude of the received signal with both beams as a function of the distance between emitter and receiver; and (**f**) directivity pattern of both beams.

The signal for the generation of the sine sweep signal was designed by modulating a sine sweep signal from 20 kHz to 50 kHz using a modulation frequency of 495 kHz. Figure 10a shows the emission signal, where the modulation shape can be observed. The received signal was a mix of the primary beam at 495 kHz and the secondary beam at low frequency produced by parametric effect. In order to distinguish the secondary beam, a band pass (5–80 kHz) filter was applied. Figure 10b shows the received signal, primary (original) and secondary (filtered) beams. The analyses of the different parametric studies with this signal were obtained by taking the amplitude value of the correlation of the received signal with the primary beam signal (high frequency) and with the expected secondary beam signal (low frequency). Figure 10c shows the correlation of the original received signal with the expected secondary beam signal. There is a clear correlation peak at the arrival time of the received signal.

Figure 10d shows the correlation amplitude behaviour of the received signal, without filtering (original) and with the band-pass filtering (secondary beam) as a function of the input signal amplitude. The data fitting to parameterizations showed that the exponent for the secondary beam is much larger than that of the primary beam, which is evidence of the non-linear effect. The exponent value of the primary beam is 0.69 ± 0.03 and for the secondary beam is 3.70 ± 0.12. We expected an exponent close to one for the primary beam, but it is slightly lower due to saturation effects of the amplifier. On the other hand, for the secondary beam, the expectation for the exponent was two, but it is larger because of the cross-correlation method used. In fact if we were directly representing the amplitude of the signal of the secondary beam filtered, the exponent would have been 1.68 ± 0.15. However, when using the correlation method for the secondary beam, following the parametric theory, the received signal is correlated with the second time derivative of the envelope squared of the emitted signal. The fact that we use the square in this process should be why the exponent is almost twice the expected value.

The evolution of both beams with the distance is shown in Figure 10e, clearly displaying the different behaviour between them. The ratio of amplitudes between the primary and secondary beam increases as a function of the distance, which is evidence of the secondary beam being generated in the medium. Parameterizations were fitted to the data obtaining a smaller exponent for the secondary beam, as expected. For the primary beam the exponent value is −0.44 ± 0.04 while for the secondary beam is −0.27 ± 0.06.

The directivity patterns shown in Figure 10f are also evidence of the parametric effect for the secondary beam since the pattern for this beam is quite similar to that of the primary beam, and more directive than the ceramic directly fed at low frequency. For instance, the open angle (FWHM) for direct feeding in the frequency range, 20–50 kHz, was 80° approximately.

### 4.2. Parametric Bipolar Pulse Signal

**Figure 11:**
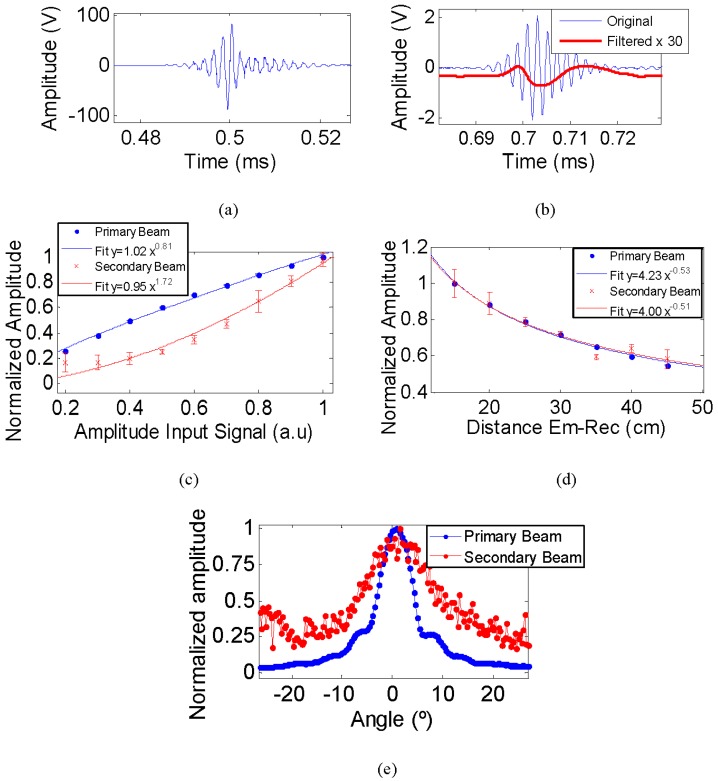
(**a**) Emitted signal for bipolar pulse generation; (**b**) received signal (blue line) and bipolar signal obtained after applying a band-pass filter (red line); (**c**) amplitude of the received (blue) and filtered signal (red) as function of input signal amplitude; (**d**) amplitude of the received signal for both beams as function of distance between emitter and receiver; and (**e**) directivity pattern of both beams.

The emission signal for achieving the parametric generation of the bipolar-shape pulse was designed by using the portable signal at 495 kHz. According to theory, the signal is set so the shape of the secondary signal matches the second time derivative of the envelope squared of the primary signal in amplitude [18]. Figure 11a shows the emitted signal shape modulated. The received signal was a mix of the primary beam at 495 kHz and the secondary beam at low frequency produced by parametric effect. In order to distinguish the secondary beam, a band pass (5–80 kHz) filter was applied. Figure 11b shows the received signal, primary (original) and secondary (filtered) beams. The secondary beam (bipolar pulse) is multiplied by a factor of 30 in order to be visible together with the original received signal (non-filtered). As expected, the secondary beam has a bipolar pulse-shape. A peak after the bipolar pulse is appreciated. That is due to the signal response of the transducer, to what can affect to the shape of the signal tail and the generation of an oscillation after the last signal pulse. The analysis of the different parametric studies with this signal were obtained by taking the amplitude value of the signal received at high frequency (primary beam) and of the bipolar pulse signal (secondary beam).

Figure 11c shows the amplitude behaviour of the received signal, without filtering (original) and with the band-pass filtering (the parametric bipolar signal) as a function of input signal amplitude. The fitting data to parameterizations showed that the exponent for the secondary beam is twice the exponent of the primary beam, representing the non-linear effect. The exponent value of the primary beam is 0.81 ± 0.03 and for the secondary beam is 1.72 ± 0.26. The exponent for the primary beam is slightly below one due to saturation effects of the amplifier. In Figure 11d the evolution of both beams with the distance can be observed. No conclusive results could be obtained from this due to the fluctuations observed in the measurements and on the detection of the bipolar secondary signal, since the exponent value for the primary beam is −0.53 ± 0.01 and for the secondary beam is −0.51 ± 0.05. The directivity pattern, shown in Figure 11e, is the best proof of the parametric effect for the secondary beam since both beams have similar directivities.

Considering all these results, the parametric generation was satisfactory validated. The large tube with aluminium backing and moulded with polyurethane EL241F, as a matching layer, showed very good characteristics for use as the transducer unit of the final array.

## 5. Future Steps

The work to follow will consist of adapting the electronics to the final transducer design to achieve the required power emission able enough to produce the parametric signals. The new electronic board will be based on the same philosophy as the previously designed electronic board [19], while adapted to the particularities of the new transducer and application. The electronics will also incorporate the functionalities for communication, configuration, control, and amplification of the signal that were already developed in previous studies [20].

Afterwards, the transducer array will be built, which will be composed of multi-emitters placed in the same line structure, which will be a long tube of aluminium that will also work as backing of the ceramics. After the characterization and final tests over longer distances are completed, the calibrator will either be used in sea campaigns for emissions from a vessel or incorporated in the KM3NeT deep-sea neutrino telescope infrastructure, in order to perform the calibration tests for the acoustic telescope systems.

## 6. Conclusions

The results obtained on the characterization of the transducers designed (piezo-ceramics with backing and matching layer) indicate that the best solution for the multi-element array calibrator is the large tube with aluminium backing and moulded with 3λ/4 of polyurethane EL241F. An increment of 9 dB after backing and moulding was appreciated on the transducer, compared to the bare ceramic, with a final sensitivity of 169 dB (re μPa/V at 1 m) at 495 kHz. The parametric emission was validated for both parametric signals generated, namely the sine sweep and the bipolar pulse; the non-linear effect of the parametric signal was verified in the studies performed, which showed higher amplitude evolution and a narrower directivity pattern. Moreover, by using the parametric technique a signal that imitates the acoustic neutrino-like bipolar pulse was achieved.

The future array of multi-elements emitting at linear or parametric modes with a high-pressure sound level will become a very versatile and powerful system, which could be used in other kinds of underwater acoustic applications, such as communication, localization, positioning and on-site sensor calibration.

## Figures and Tables

**Table 1:** Acoustic properties of the matching layer (ML) materials RoyaPox 511 and EL214F, and thickness (mm) obtained for accomplishing *λ*/4 or 3*λ*/4 of the emitted wavelength at the ceramic resonance frequency.

ACOUSTIC PROPERTIES	Large Tube M.L RoyaPox 511	Large Tube M.L EL214F	Small Tube M.L EL214F
c_L_ (m/s)	2602	1600	1600
Z (MRayls)	2.82	1.857	1.857
ρ (kg/m^3^)	1050	1160.7	1160.7
Frequency (kHz)	480	510	960
*λ*/4 Thickness (mm)	1.3	0.78	0.42
3*λ*/4 Thickness (mm)	4.0	2.4	1.3
